# Autistic traits and obsessive-compulsive personality traits in OCD patients

**DOI:** 10.1186/s43045-022-00213-0

**Published:** 2022-06-13

**Authors:** A. Abd Elgawad, A. Elbatrawy, E. Shorub, M. Ramadan, H. Elkhatib

**Affiliations:** 1 Neuro-Psychiatry, Faculty of Medicine, Ain Shams University, Cairo, Egypt; 2 Menofia University, Shibin el Kom, Egypt; 3 Faculty of Medicine, Misr University for Science and Technology, October City, Egypt

**Keywords:** Obsessive-compulsive disorder, Yale-Brown Obsessive-compulsive Scale, Autism spectrum quotient, Structured Clinical Interview for DSM-IV (SCID-II), Obsessive-compulsive personality, Egypt

## Abstract

**Background:**

Studies have reported a high prevalence of autism spectrum disorder in young people with obsessive-compulsive disorder with a negative effect on psychosocial functioning. However, the extent to which autism spectrum disorder and obsessive-compulsive personality disorder traits overlap and by inference, the extent to which these separately classified Diagnostic and Statistical Manual of Mental Disorders disorders five (obsessive-compulsive personality disorder, personality disorder, autism spectrum disorder, neurodevelopmental disorder) may share a nosological relationship has not so far been systematically investigated in clinical samples.

This study is done to detect the frequency of obsessive-compulsive personality traits and autistic traits in a sample of patients with obsessive-compulsive disorder.

**Results:**

Results revealed that younger patients had significantly more severe and extreme obsessive-compulsive disorder scores. Moreover, Structured Clinical Interview for DSM-IV (SCID-II) and Autism Spectrum Quotient analysis revealed that younger patients had a significantly higher prevalence of personality traits and autistic traits respectively. Statistical significance as many patients with severe and extreme Yale-Brown Obsessive-Compulsive Scale showed criteria of obsessive-compulsive personality disorder. No association of statistical significance was found between obsessive-compulsive disorder severity and autistic trait presence. On the contrary, statistical significance was found between autistic traits and obsessive-compulsive personality disorder.

**Conclusions:**

There is the presence of comorbidity of obsessive-compulsive personality traits and autism spectrum traits in obsessive-compulsive disorder patients. Obsessive-compulsive personality traits prevalence in obsessive-compulsive disorder patients was higher than in autistic traits. Several factors of genetic predisposition, environmental factors like education and marital status, employment, and intrinsic factors as age of patients all exhibited a pivotal role in obsessive-compulsive disorder prevalence and severity.

## Background

Some behaviors associated with obsessive-compulsive disorder such as anxiety, repetitive behaviors, and social problems are also typical of autism spectrum disorder. While the appearance of autism spectrum disorder and obsessive-compulsive disorder may be similar on the surface, the processes that drive these behaviors are quite different; a common though often overlooked comorbidity in treatment-seeking obsessive-compulsive disorder patients.

There is a high prevalence of autism spectrum disorder in young people with obsessive-compulsive disorder with a negative effect on psychosocial functioning as many studies showed. However, the extent to which autism spectrum disorder and obsessive-compulsive personality disorder traits overlap and by inference, the extent to which these separately classified Diagnostic and Statistical Manual of Mental Disorders disorders five disorders (obsessive-compulsive personality disorder, personality disorder, autism spectrum disorder, neurodevelopmental disorder) may share a nosological relationship has not so far been systematically investigated in clinical samples [1].

Studies suggest a possible neurodevelopmental etiology for this comorbid subgroup. Other signs of an altered neurodevelopmental trajectory, such as traits or symptoms of tic disorder, autism spectrum disorder, and attention deficit hyperactivity disorder, may also be observed in patients with obsessive-compulsive disorder and their family members, hinting at the possibility that heritable neurobehavioral mechanisms contribute to the expression of at least some forms of obsessive-compulsive disorder.

## Objectives

To detect the frequency of obsessive-compulsive personality traits and autistic traits in a sample of patients with OCD attending Menoufia Mental Health and Addiction Treatment Hospital general adult psychiatry outpatient clinics.

## Methods

### Study design

This is an observational cross-sectional study.

### Study place

Menoufia, Shebin Al-kum Mental Health and Addiction Treatment Hospital general adult outpatient clinics.

### Sample population

Patients diagnosed with obsessive-compulsive disorder (OCD), attend general adult psychiatry outpatient clinics in Menoufia Mental Health and Addiction Treatment Hospital.

### Sample size

Using PASS Program for sample size calculation and assuming the proportion of OCD patients suffering from OCPD = 30% with a 10% margin of error and at a 90% confidence level, a sample size of 60 patients was calculated.

### Sample selection

A convenient sample of 60 patients was included in the study. Participating patients were of both genders.

### Inclusion criteria

Age was between 18 and 60 years. Patients giving consent and diagnosed with OCD attending general adult psychiatry outpatient clinics in Menoufia Mental Health and Addiction Treatment Hospital. Both males and females will be included.

### Exclusion criteria

Patients refused to participate in the study. Known psychiatric illness and substance use disorders other than OCD. Any current chronic medical illness.

Current medication intake or a substance, e.g., cortisone, appetite suppressants

### Assessment and procedures

After explaining the purpose of the study, patients were informed that participation was voluntary and that any patient can exit from the study at any time without giving justification. Subjects of the study were anonymous and the results of the study would only be used for scientific purposes. Written informed consent was obtained.

Patients attending general adult psychiatry outpatient clinics in Menoufia Mental Health and Addiction Treatment Hospital during the study period from the start of the clinical sample collection stage in July 2020 until the completion of the sample in February 2021 were interviewed clinically for diagnosing the OCD symptoms according to the Diagnostic and Statistical Manual of Mental Disorders, 4th Edition (DSM IV) [2], and OCD symptom by the Y-BOCS symptom severity scale and symptom checklist interview by an expert psychiatrist.

The total number of cases screened was 110 patients comorbid with other psychiatric disorders to find including 60 patients eligible for this study with compensating [3] dropped out cases due to refusal to continue completing the scales to the end excusing by time, poor literacy, being from the low socioeconomic strata, or minority race/ethnicity. Participants’ poor health/general condition status. Data were collected from the patients.

### Study procedures

All patients were subjected to the following:

Full personal history, full medical, and neurological history including a detailed history of substance use disorders, detailed history of psychiatric disorders, detailed history of a medical disorder, and current medication. A general and neurological examination.

Patients fulfilling inclusion criteria were subjected to the following:

Clinical interview for diagnosing the OCD symptoms according to the Diagnostic and Statistical Manual of Mental Disorders, 4th Edition (DSM-IV), Confirming Diagnoses using Structured Clinical Interview for DSM-IV (SCID-I) and excluding any other known psychiatric illness and substance use disorders [3].

Assessing OCD symptoms using a Y-BOCS symptom checklist interview and the Y-BOCS symptom severity scale by an expert psychiatrist in general adult psychiatry outpatient clinics [4].

Obsessive-compulsive personality profile was evaluated using Structured Clinical Interview for DSM-IV (SCID-II) [5].

Autistic traits assessed by using self-administered Autism Spectrum Quotient-Arabic version (AQ) [6].

### Data analysis

The collected data was revised, coded, and tabulated using the Statistical Package for Social Science (IBM Corp. Released 2017. IBM SPSS Statistics for Windows, Version 21.0. Armonk, NY: IBM Corp.) [7].

### Descriptive statistics

Mean, standard deviation (±SD) for numerical data, frequency, and percentage of non-numerical data.

### Analytical statistics

An independent *t* test was used to assess the statistical significance of the difference between the two study group means. One-way ANOVA test was used for three or more groups of data to gain information about the relationship between the dependent and independent variables through comparison of mean values. The chi-square test was used to examine the relationship between two qualitative variables.

*P* value: level of significance: *P* > 0.05: non-significant, *P* ≤ 0.05: significant, *P* ≤ 0.01: highly significant.

## Results

### Sample description

The current study included 60 eligible patients diagnosed with OCD by SCID-II. The mean age of included patients was 37.5 ± 13 years. Seventy percent (*n* = 42) of included patients were males and 30% were females (*n* = 18).

Regarding marital status, 28.3% of included patients were single (*n* = 17), 56.7% were married (*n* = 34), 6.7% were divorced (*n* = 4), and 8.3% were widowed (*n* = 5).

Regarding occupational status, of the included patients, 25% were employed (*n* = 15), and 75% were unemployed (*n* = 45). Regarding the educational level, 25% patients were illiterate (*n* = 15), 16.6% (*n* = 10) had preparatory education, 31.7% (*n* = 19) had secondary education, and 26.7% (*n* = 16) had high education.

Family history of psychiatric diseases was present in 56.7% (*n* = 34) patients, and further assessment of family history was subdivided into family history of psychosis in 33.3% of patients (*n* = 20), family history of neurosis in 23.3% of patients (*n* = 14), and negative family history of psychiatric diseases in 43.3% patients (*n* = 20) (Table 1).

**Table 1:**
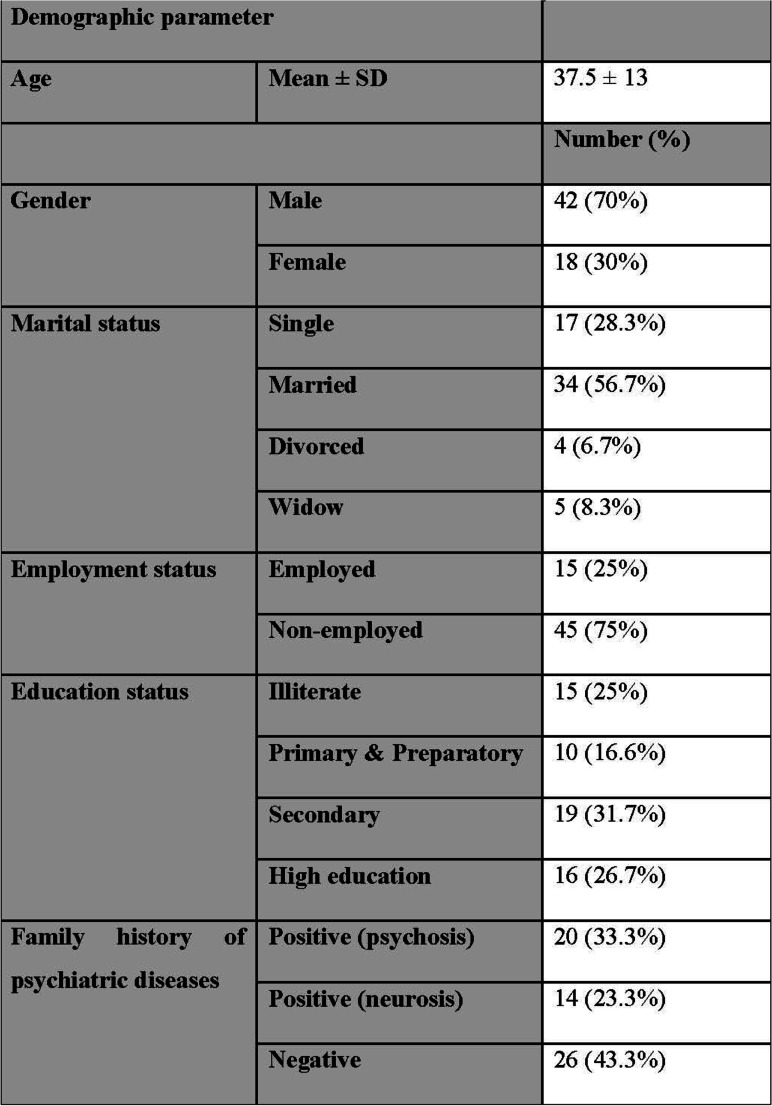
Demographic data of included patients

### Assessment of Y-BOCS, SCID-II, and AQ results in comparison to different demographic data

Assessment of Y-BOCS results about age revealed that younger patients had significantly more severe and extreme OCD scores. Moreover, SCID-II and AQ analyses about age revealed that younger patients had a significantly higher prevalence of personality traits and autistic traits respectively (Table 2).

**Table 2:**
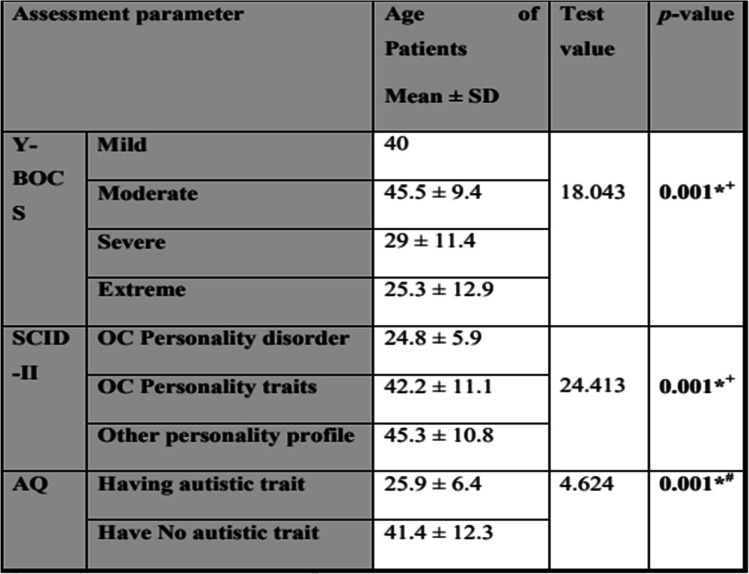
Assessment of Y-BOCS, SCID-II, and AQ results in comparison to age

*Y-BOCS* Yale-Brown Obsessive Compulsive Scale, *SCID* Structured Clinical Interview for DSM-IV Disorders, *AQ* Autism Spectrum Quotient

^+^Using one-way ANOVA test

^#^Using independent *t* test, *p value ≤ 0.05 is significant, *p* value ≤ 0.01 is highly significant

### Inter-comparison of Y-BOCS, SCID-II, and AQ results in included patients

a-Comparison of Y-BOCS with SCID-II revealed statistical significance as many patients with severe and extreme Y-BOCS scale showed criteria of OC personality disorder (Table 3).

**Table 3:**
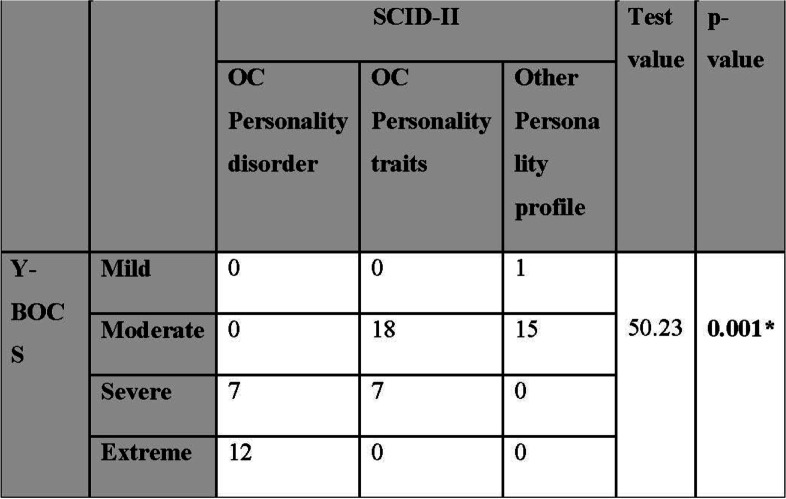
Inter-comparison of Y-BOCS and SCID-II results in included patients

*Y-BOCS* Yale-Brown Obsessive Compulsive Scale, *SCID* Structured Clinical Interview for DSM-IV Disorders

Using the chi-square test, *p value ≤ 0.05 is significant, *p* value ≤ 0.01 is highly significant

b-Comparison of Y-BOCS with AQ revealed no association of statistical significance between OCD severity and autistic trait presence (Table 4).

**Table 4:**
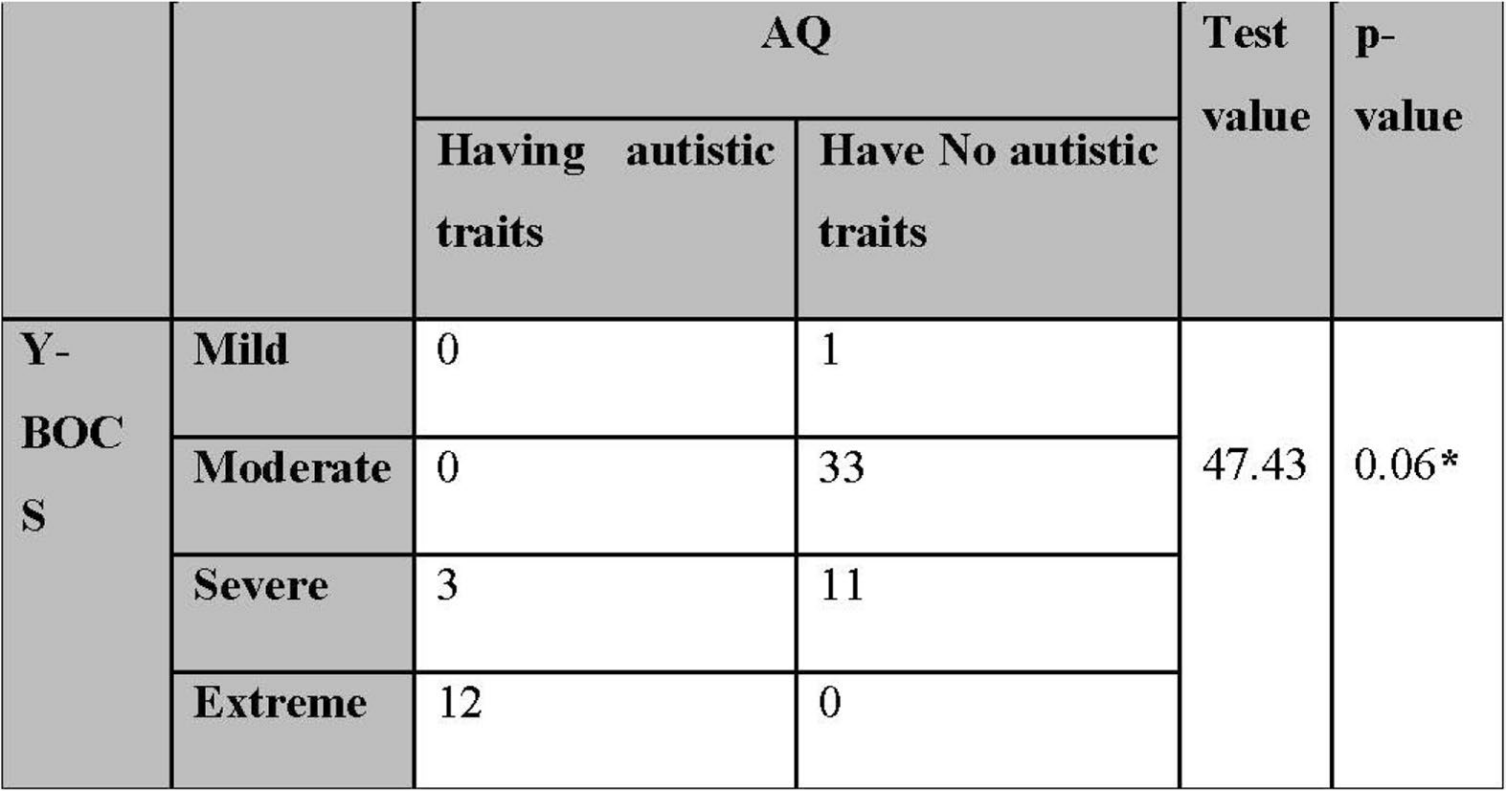
Inter-comparison of Y-BOCS and AQ results in included patients

*Y-BOCS* Yale-Brown Obsessive Compulsive Scale, *AQ* Autism Spectrum Quotient

Using the chi-square test, *p value ≤ 0.05 is significant, *p* value ≤ 0.01 is highly significant

### Comparison of AQ with SCID-II results

Comparison of AQ with SCID-II results revealed statistical significance as many patients who had autistic traits showed criteria of OC personality disorder (Table 5).

**Table 5:**
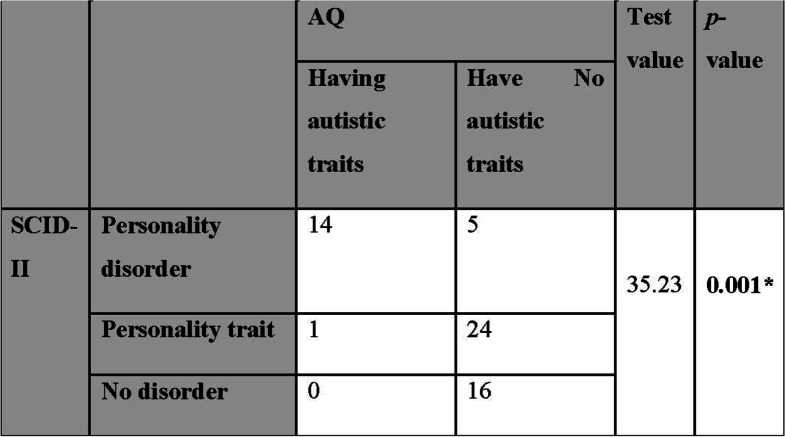
Comparison of AQ with SCID-II results

*SCID* Structured Clinical Interview for DSM-IV Disorders, *AQ* Autism Spectrum Quotient

Using the chi-square test, *p value ≤ 0.05 is significant, *p value ≤ 0.05 is significant, *p* value ≤ 0.01 is highly significant

## Discussion

Whether ASD or AS traits may have significant impacts on clinical and psychosocial features as well as a long-term treatment outcome in adult OCD patients has been investigated in few studies. In the present study, we sought to detect the frequency of autistic traits and obsessive-compulsive personality traits in a sample of patients with OCD. The current study included 60 eligible patients diagnosed with OCD by SCID-II. The mean age of included patients was 37.5 ± 13 years. A significant male predominance of 70% (*n* = 42) of included patients was noticed while 30% were females (*n* = 18). This can be due to cultural beliefs that restrict female participation in psychological based studies.

Contrary to our results, the study population was reported in different studies. In a study done by Gadelkarim et al. [8] included 67 individuals (52.2% female and 48.8% males with mean age of 44.5 years, SD ± 11.47).

Also, Wikramanayake et al. [9] included 73 adult outpatients with DSM-IV OCD with a mean age of 44.7 years and slight female predominance (53.4%). Using SCID-II in the assessment of personality profiles revealed that 31.7% of patients (*n* = 19) had OC personality disorder, 41.7% of patients (*n* = 25) had OC personality traits, and 26.7% of patients (*n* = 16) had no OC personality traits.

Gordon et al. [10] in their study reported that of the 189 participants with a diagnosis of OCD, 104 (55%) did not meet DSM-IV criteria for OCPD, while 85 (45%) met the criteria for OCD and OCPD. Moreover, OCPD was significantly associated with OCD. Results from this study demonstrate a higher rate of co-occurring OCD with OCPD than our study. This is probably because all OCD patients were assessed using the SCID OCPD module, not only those who scored positive on the Axis II SCID screener.

Mancebo et al. [11] concluded that similarities in phenomenology can make it difficult to differentiate between OCPD and OCD, giving the example of excessive list-making, which can count as a preoccupation with detail in DSM-IV OCPD and may be considered a compulsion if it is repetitive, time-consuming, and distressing. Again, they suggest that perfectionism, while a criterion for OCPD, is also a symptom of OCD if it involves order, symmetry, and arranging. Phenomenological differences occurred between OCD and OCPD, such as the ego-dystonic nature of OCD symptoms which differs from the ego-syntonic nature of OCPD characteristics, also the fact that intrusive thoughts and repetitive behaviors are not typically experienced by individuals with OCPD as they are in OCD [12].

Our study assessment of OCD symptom severity using the Y-BOCS scale revealed that 1 patient had mild symptoms, 33 patients had moderate symptoms, 14 patients had severe symptoms, and 12 patients had extreme symptoms. Certain OC symptoms were more commonly endorsed than others within the OCD sample when patients included in the study underwent a YBOCS symptom checklist. Similar to the current study, Mito et al. (2014) reported that aggressiveness followed by contamination and symmetry and exactness were the most commonly observed obsessions while cleaning, washing, and checking were the most common compulsions.

On the contrary, Gordon et al. [10] and Gadelkarim et al. [8] reported that the most common observed obsessions were contamination followed by hoarding and aggression while, the most common observed compulsions were cleaning, checking, and repeating. The results of the current study given the following studies agreed that a combination of OCD with autistic traits is slightly associated with higher severity of OCD symptoms.

Gadelkarim et al. [8] found that assessment of OCD symptom severity using the Y-BOCS scale revealed that patients with OCDP had higher Y-BOCS compared to patients without OCPD but with no statistical significance. Similarly, Mito et al. (2014) reported a non-significant higher Y-BOCS score in combined OCD/ASD patients compared to OCD/non-ASD patients.

Also, Wikramanayake et al. [9] reported a borderline significant (*p* value = 0.054) increase in Y-BOCS scores in patients with combined OCD/ASD patients compared to OCD/non-ASD patients.

Regarding the relation between OCPD and ASD, in contrast to our results, Gadelkarim et al. [8] concluded that approximately half (54.2%) of those diagnosed with OCPD were also found to meet diagnostic criteria for ASD. This conflicting result may be due to high symptom overlap in patients with high OCD scores in the current study.

Comparison of Y-BOCS with SCID-II revealed statistical significance as many patients with severe and extreme Y-BOCS scale showed criteria of OC personality disorder. Our assessment using the Y-BOCS symptom severity scale of patients’ demographic data revealed that younger patients had significantly more severe and extreme OCD scores.

In contrast to our study, Gadelkarim et al. [8] did not report any significant association between patients’ age and severity of OCD.

In the current study, the Autism Spectrum Quotient assessment of included patients revealed that 25% of patients (15) screened as having autistic traits while 75% of patients (45) have no any autistic traits. Besides, younger patients had a significantly higher prevalence of autistic traits. Assessment of AQ in comparison to other demographic data revealed there was no significant association between AQ scores and any of the demographic variables. The current study results were conflicting with data from previous literature.

Chaste and Leboyer, [13] reported the pivotal role of genetics in the development of autistic disorders. Lord et al. [14] showed that environmental factors had a major role in autism development. Maternal age, birth weight, childhood nutrition, and social factors like education all participate in the pathogenesis of autistic disorders. A possible explanation for the discrepancy between our results and the mentioned research can be due to different research aims needed to demonstrate risk factors behind shred disease neurodevelopmental basis. In contrast to our results, concerning family history and probability of hereditary role, there is emerging evidence of shared neurobiology among OCPD and ASD. Research on twins suggests that OCPD is highly heritable Gjerde et al. [15], and there is evidence that OCPD, OCRDs, and ASD cluster are not only found in the same patients (Hofvander et al. [16]) but also in their family members (Bienvenu et al. [17]).

These studies suggest that these disorders may share genetic factors in their etiology. Hollander et al. [18] found that the occurrence of obsessive-compulsive traits or disorders in the parents of autistic children is significantly more likely if autistic children have a high occurrence of repetitive behaviors, additionally indicating a possible role for compulsivity as a neuropsychological factor mediating familial risk across OCD, OCPD, and ASD diagnoses, supporting the above hypothesis.

In a study by Meier et al. [19] to clarify the patterns of comorbidity, longitudinal risks, and shared familial risks between OCD and autism spectrum disorders. The risk of a comorbid diagnosis of OCD in individuals with autism spectrum disorder and aggregation of autism spectrum disorders in offspring of parents with OCD were increased. Individuals first diagnosed with autism spectrum disorders had a 2-fold higher risk of a later diagnosis of OCD whereas individuals diagnosed with OCD displayed a nearly 4-fold higher risk to be diagnosed with autism spectrum disorders later in life. Therefore, the high comorbidity, sequential risk, and shared familial risks between OCD and autism spectrum disorders are suggestive of partially shared etiological mechanisms between these mental disorders.

Finally, the current study revealed that the comparison of AQ with SCID-II results showed statistical significance as most patients who had autistic traits showed criteria of OC personality disorder. However, there was no statistically significant association between OCD severity and autistic trait.

In partial agreement with our study, Gadelkarim et al. [8] reported that the severity of OCPD traits and ASD traits showed a significant positive correlation as (50.75%) of OCD patients represented a high overall frequency of ASD traits.

Assessing ASD traits using the Autism Spectrum Quotient (AQ) by Hironori et al. [20] in 81 patients with OCD, a substantial proportion of OCD patients (35%) were demonstrated to have clinically significant ASD traits according to AQ. OCD subjects with higher ASD trait (ASD+ group) were characterized by a shorter duration of education, lower Global Assessment of Functioning (GAFS), higher proportion of subjects with poor insight or with tick-related OCD than the other group. This may be due to methodological differences in the characteristics of the participants besides the different assessment procedures of ASD traits; most of the subjects were introduced because they had previously been assessed as treatment-refractory enough to receive specialized treatments for OCD, treatment-refractory patients are different types of the patient population differ from our study population.

Wikramanayake et al. [9] found a high prevalence of ASD traits (47%) and ASD diagnosis (29%) in the OCD sample.

OCD patients with autistic traits were more severely symptomatic on the Y-BOCS, and there was a positive correlation between OCD and ASD scores. This finding replicates that of Hironori et al. [20]. It is commonly found that comorbid disorders predict a greater severity of index illness. Fineberg et al. [21] and this finding may therefore simply represent a non-specific effect reflecting the burden of having an additional mental health diagnosis.

## Study limitations

### Strengths

There is relatively adequate patient sample in the studied disorder. There are multiple valid self-administrated tools for gathering data with guiding patients according to their educational level. This study is considered a newly admitted academic article in this clinical era. Due to a small number of clinical researches in this quota of disorders with high symptom overlap, good interpretations of findings that can be generalized if the sample size is representative of the study population.

### Limitations

Insufficient data was collected from the patients’ charts. The study underwent during a COVID-19 pandemic; so, it took a long time because general clinic patients’ flow rate was reduced to half of its capacity and there was a lack of non-verbal communication due to discussion 106 of face mask wearing for both psychiatrist and study subjects. One limitation is that the AQ scale is a self-report open to the bias of subjectivity. There is probably a need for developing scales that are still easy to use and are more objective in the future.

## Conclusions

Our data demonstrated that there is the presence of comorbidity of OCP traits and AS traits in OCD patients. OCP traits prevalence in OCD patients was higher than in autistic traits. Several factors of genetic predisposition, environmental factors like education and marital status, employment, and intrinsic factors as age of patients all exhibited a pivotal role in OCD prevalence and severity.

## Data Availability

Applicable

## Abbreviations

ASD: Autism spectrum disorder
AD: Autistic disorder
OCD: Obsessive-compulsive disorder
OCP: Obsessive-compulsive personality
